# Targeting the Mitochondrial Protein VDAC1 as a Potential Therapeutic Strategy in ALS

**DOI:** 10.3390/ijms23179946

**Published:** 2022-09-01

**Authors:** Anna Shteinfer-Kuzmine, Shirel Argueti-Ostrovsky, Marcel F. Leyton-Jaimes, Uttpal Anand, Salah Abu-Hamad, Ran Zalk, Varda Shoshan-Barmatz, Adrian Israelson

**Affiliations:** 1 Department of Life Sciences and the National Institute for Biotechnology in the Negev, Ben-Gurion University of the Negev, Beer-Sheva 84105, Israel; 2 Department of Physiology and Cell Biology, Faculty of Health Sciences and The Zlotowski Center for Neuroscience, Ben-Gurion University of the Negev, Beer-Sheva 84105, Israel; 3 Department of Stem Cell and Regenerative Biology, Sherman Fairchild, Harvard University, 7 Divinity Ave., Cambridge, MA 02138, USA; 4 Ilse Katz Institute for Nanoscale Science & Technology, Ben-Gurion University of the Negev, Beer-Sheva 84105, Israel

**Keywords:** VDAC1, ALS, mutant SOD1, mitochondria, apoptosis, misfolded proteins

## Abstract

Impaired mitochondrial function has been proposed as a causative factor in neurodegenerative diseases, including amyotrophic lateral sclerosis (ALS), caused by motor neuron degeneration. Mutations in superoxide dismutase (SOD1) cause ALS and SOD1 mutants were shown to interact with the voltage-dependent anion channel 1 (VDAC1), affecting its normal function. VDAC1 is a multi-functional channel located at the outer mitochondrial membrane that serves as a mitochondrial gatekeeper controlling metabolic and energetic crosstalk between mitochondria and the rest of the cell and it is a key player in mitochondria-mediated apoptosis. Previously, we showed that VDAC1 interacts with SOD1 and that the VDAC1-N-terminal-derived peptide prevented mutant SOD1 cytotoxic effects. In this study, using a peptide array, we identified the SOD1 sequence that interacts with VDAC1. Synthetic peptides generated from the identified VDAC1-binding sequences in SOD1 directly interacted with purified VDAC1. We also show that VDAC1 oligomerization increased in spinal cord mitochondria isolated from mutant SOD1^G93A^ mice and rats. Thus, we used the novel VDAC1-specific small molecules, VBIT-4 and VBIT-12, inhibiting VDAC1 oligomerization and subsequently apoptosis and associated processes such as ROS production, and increased cytosolic Ca^2+^. VBIT-12 was able to rescue cell death induced by mutant SOD1 in neuronal cultures. Finally, although survival was not affected, VBIT-12 administration significantly improved muscle endurance in mutant SOD1^G93A^ mice. Therefore, VBIT-12 may represent an attractive therapy for maintaining muscle function during the progression of ALS.

## 1. Introduction

Amyotrophic lateral sclerosis (ALS) is a fatal neurodegenerative disease caused by the degeneration of upper and lower motor neurons in the brain and spinal cord, leading to muscle weakness, paralysis, and death. Most ALS cases do not follow a Mendelian inheritance, referred to as sporadic ALS (sALS), but around 10% is inherited with familial ALS characteristics (fALS) [1]. About 20% of fALS cases are associated with mutations in Cu/Zn superoxide dismutase (SOD1) and represent the second most common form of fALS. Over 180 different mutants with and without dismutase activity have been characterized in humans [2]. In affected tissues, the toxic effects of SOD1 mutants are related to the formation and accumulation of misfolded SOD1 aggregates and their binding to the endoplasmic reticulum (ER) and the mitochondrial surface, leading to morphological degeneration and malfunctioning of the organelles [3,4].

Initial pathogenic factors proposed in ALS include induction of ER stress [5,6], reduction of proteasome activity [7,8], reduction of autophagy [9], aggregation [10], oxidative damage [11], and Ca^2+^-mediated excitotoxicity [12]. Several studies have also implicated mitochondria as targets for ALS toxicity [13,14]. Mitochondria are implicated in several cell processes such as cellular energy generation, metabolism, cell proliferation, differentiation, and apoptosis, among many others [15]. Therefore, it is not surprising that mitochondrial dysfunction is associated with various human diseases [16], including a central role in ALS neuropathology. For instance, alteration of ATP production, maintenance, and oxygen consumption is correlated with altered CHCHD10 gene expression, leading to “mitochondrial DNA breakage” [17]. The shortage of maintenance of the cristae architecture of the mitochondria has been associated with disruption in CHCHD3 and OPA1 interactions [18].

A key regulator of mitochondrial function is the voltage-dependent anion channel 1 (VDAC1), a multi-functional protein located on the outer mitochondrial membrane (OMM). VDAC1 serves as a mitochondrial gatekeeper, controlling the metabolic and energetic crosstalk between mitochondria and the rest of the cell, and it is also one of the key proteins in mitochondria-mediated apoptosis [19,20,21,22].

Three isoforms of VDAC (VDAC1, VDAC2, and VDAC3) have been identified in mammals sharing several structural and functional properties [23,24]. Of the three isoforms, VDAC1 is the most abundant and best studied. VDAC1 stands at the crossroads between mitochondria-mediated energy and metabolism, and apoptosis [25].

The positioning of VDAC1 at the OMM also enables its interaction with proteins that mediate and regulate mitochondrial functions with other cellular activities. Indeed, VDAC1 is considered a hub protein, interacting with over 100 proteins [25]. The VDAC1 interactome includes proteins involved in metabolism, apoptosis, signal transduction, anti-oxidation, DNA- and RNA-associated proteins, and more [20,26,27,28,29]. VDAC1 interacts with proteins involved in energy homeostasis, such as adenine nucleotide translocase (ANT), tubulin, glycogen synthase kinase (GSK3), creatine kinase, and hexokinase (HK), and with apoptosis-regulating proteins such as Bax, Bcl-2, and Bcl-xL [20,25,30].

Mutant SOD1 has been found to specifically interact with VDAC1, leading to inhibition of channel conductance that limits the transport of molecules and particles across the OMM, thus, compromising the energy supply to the motor neurons [31]. This binding has been recently characterized and shown to occur via interaction of misfolded SOD1 with the VDAC1 N-terminal domain [32]. Moreover, in NSC-34 cells expressing mutant SOD1, post-translational modifications in VDAC1 residues have been reported, suggesting the appearance of important changes in the structure of the VDAC1 channel and, therefore, to the bioenergetic metabolism of ALS motor neurons [33].

VDAC1 is involved in dysregulated metabolism and cell death, inflammation, and fibrosis associated with cancer and several other diseases [34]. We recently found that VDAC1 is over-expressed not only in cancer [22,35,36,37], but also in several diseases such as Alzheimer’s disease (AD) [38,39,40], type 2 diabetes (T2D) [41,42,43], and autoimmune diseases such as lupus [44] and colitis [45].

As overexpressed VDAC1 triggers cell death via its oligomerization [46,47,48], we developed new molecules, e.g., VBIT-4 and VBIT-12, that prevent this oligomerization and subsequent apoptosis [49], and inhibit VDAC1 conductance, as was also validated in T2D [43], lupus [44], and colitis [45] mouse models.

In this study, we identified the SOD1 sequence interacting with VDAC1, found to be localized on the protein surface, and the identified VDAC1-binding sequences formed as synthetic peptides directly interact with purified VDAC1. We show that inhibiting the oligomerization of VDAC1 using VBIT-12 was able to rescue cell death induced by mutant SOD1 in motor-neuron-like NSC-34 cells. Moreover, administration of VBIT-12 to mutant SOD1^G93A^ mice significantly improved muscle endurance during the disease course. Therefore, VBIT-12 may represent an attractive therapy for maintaining muscle function during the progression of ALS.

## 2. Results

### 2.1. Identification of the VDAC1 Interaction Site in SOD1

**Figure 1:**
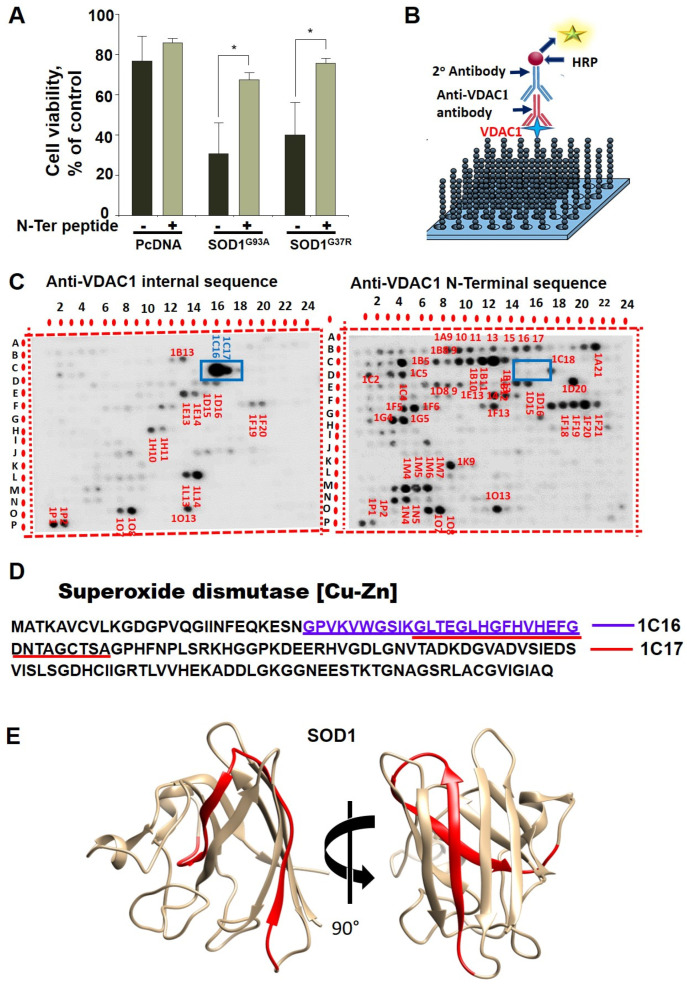
Identification of VDAC1 interacting sites in SOD1. (**A**) SH-SY5Y cells (4.5 × 10^4^ cells/well in 24-well plates) were transfected with an empty plasmid or a plasmid encoding for mutant SOD1^G93A^ or SOD1^G37R^. Then, 24 h post-transfection, the cells were incubated for 5 h with (10-20)N-Ter-Antp peptide (20 µM) and then analyzed for cell death. The results are from triplicates of different biological repeats (n = 3) and are presented as the means ± SEM. * *p* < 0.05. (**B**) Schematic presentation of peptide arrays and detection of VDAC1-interacting peptides. (**C**) Glass-bound peptide array consisting of 768 overlapping peptides derived from 19 VDAC1-interacting proteins were incubated for 4 h with purified VDAC1 (64 nM) and then blotted with anti-VDAC1 antibodies against an internal sequence (1:5000) or with antibodies against the VDAC1-N-terminus, followed by incubation with HRP-conjugated anti-mouse IgG and detection using a chemiluminescence kit. Dark spots represent binding of VDAC1 to peptides derived from VDAC1-interacting proteins. Spots 1C16 and 1C17 (squared) represent SOD1-derived peptides interacting with VDAC1 as detected with anti-VDAC1 antibodies directed to an internal sequence, but not against the N-terminus. (**D**) SOD1 sequence with the two overlapping interacting peptides is indicated. (**E**) The 3D structure of the SOD1 (PDB_ID 1PU0) with the identified peptide localization indicated (in red ribbon). Images were prepared with UCSF-Chimera [50].

As we have recently demonstrated, VDAC1 binding to mutant SOD1 involves the VDAC1 N-terminal domain [32]. In order to test whether a VDAC1-derived N-terminal peptide can rescue mutant SOD1 toxicity, we expressed two different SOD1 mutants (SOD1^G93A^ or SOD1^G37R^) in SH-SY5Y neuronal cells. These expressed SOD1 mutant proteins decreased cell survival compared to control cells. However, incubation with VDAC1-derived (10–20)-N-Ter-Antp peptide, rescued mutant SOD1-induced toxicity increasing SH-SY5Y cell survival (Figure 1A). It is important to note that in this peptide, the last six residues from the C-terminus of the N-terminal peptide were removed (∆21–26), including the GXXXG motif; therefore, it lost its cell death activity [32].

To identify the possible VDAC1 interaction site(s) in SOD1, we designed glass-bound peptide arrays consisting of 768 peptides derived from 19 selected VDAC1-interacting proteins, including peptides derived from SOD1. The proteins were selected from over 100 proteins interacting with VDAC1 and include mitochondrial-, cytoskeletal-, cell division-, cell signaling-, and apoptosis-related proteins [25,36]. Each peptide was composed of 25 amino acids, with several amino acids overlapping with the following peptide. The peptide array was incubated with purified VDAC1 and then with anti-VDAC1 antibodies against the VDAC1 internal sequence or against the VDAC1 N-Terminus, followed by the HRP-conjugated secondary antibody that was detected by the chemiluminescence reaction product (Figure 1B). The VDAC1-interacting peptides derived from the 19 VDAC1-interacting proteins are shown with which spots 1C16 and 1C17 are derived from SOD1 (Figure 1C). The peptides sequences represented by spots 1C16 and 1C17 are overlapping over 18 of the 25 amino acids. The VDAC1 interaction with IC16 yielded a bigger spot, suggesting higher affinity of VDAC1 to this sequence. The VDAC1 interaction with the two peptides was detected using antibodies against a VDAC1 internal sequence, but not with antibodies against the VDAC1 N-terminus (Figure 1C). This suggests that the N-terminus domain of VDAC1 is bound to the SOD1-derived peptides and, therefore, is not available for interaction with the antibody directed to the VDAC1-N-terminal domain.

**Figure 2:**
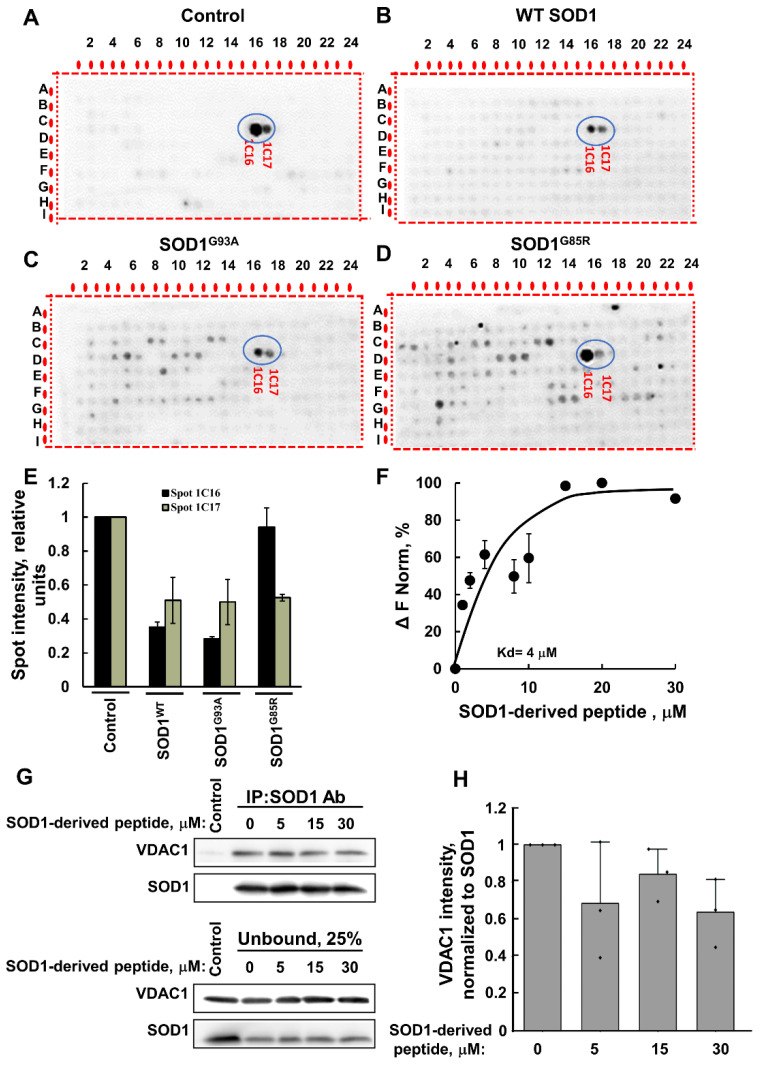
VDAC1 interaction with SOD1-derived peptides is reduced by WT and mutant SOD1. VDAC1 (64 nM) was pre-incubated for 1 h without (**A**) or with 160 nM of SOD1^WT^ (**B**), SOD1^G93A^ (**C**), or SOD1^G85R^ (**D**), followed by incubation for 4 h with the peptide array and then blotted with anti-VDAC1 antibodies, as in Figure 1C. SOD1-derived sequences are circled. (**E**) The intensity of the peptide spots 1C16 and 1C17 (circled) was analyzed using Image J. (**F**) Fluorescently labeled purified VDAC1 (138 nM) was incubated with the SOD1-derived (1C16) peptide (1–30 µM) for 30 min at 37 °C; then, MST analysis was performed, and the revealed *K*d is indicated. (**G**) Pull down with anti-SOD1 antibody was performed by incubating recombinant VDAC1 and SOD1 proteins (2 µg each) with increasing concentrations of SOD1-derived peptide (0, 5, 15, and 30 µM). Immunoblotting was used to determine the levels of SOD1^G93A^ and VDAC1 in the bound and unbound fraction of each immunoprecipitation experiment. Data indicate one representative membrane out of 3 independent experiments. (**H**) Quantification of immunoblotting bands with ImageJ software. Quantification analysis of the relative VDAC1 intensity was performed with one-way ANOVA. Bars represent mean ± SEM.

The localization of these peptides in the SOD1 linear sequence (Figure 1D) and in the SOD1 3D structure (prepared using UCSF Chimera) [50] is indicated (Figure 1E). The interaction of VDAC1 with wild type (WT) and mutant SOD1 was also analyzed indirectly. Pre-incubation of VDAC1 with purified SOD1^WT^ or two different mutants, the dismutase inactive SOD1^G85R^, or dismutase active SOD1^G93A^, decreased the VDAC1 interaction with both SOD1-derived peptides presented in spots 1C16 and 1C17 (Figure 2A–D). Although the interaction of VDAC1 with the 1C17 peptide was inhibited by pre-incubation with SOD1^WT^, SOD1^G93A^, and SOD1^G85R^ at a similar level, SOD1^G93A^ prevented the interaction of VDAC1 with the 1C16 peptide better than SOD1^WT^ and SOD1^G85R^ (Figure 2E).

Next, to validate the results, we used a synthetic peptide representing the SOD1-derived VDAC1-binding peptide (1C16) and tested its direct interaction with purified VDAC1. Fluorescently labeled VDAC1 was incubated with different concentrations of the peptide, and their interaction as monitored by the MST analysis revealed an interaction with binding affinities (*K*d) of 4 µM (F).

Moreover, the interaction of the SOD1-derived peptide with VDAC1 was supported by immunoprecipitation of the recombinant mutant SOD1^G93A^ with anti-SOD1 antibodies. Incubation of the SOD1-derived peptide with purified VDAC1 reduced the SOD1–VDAC1 interaction as measured by the decrease in VDAC1 co-precipitated with SOD1 (Figure 2G,H).

### 2.2. VBIT-12 Rescued Cell Death Induced by Mutant SOD1 in NSC-34 Motor-Neuron-like Cells

**Figure 3:**
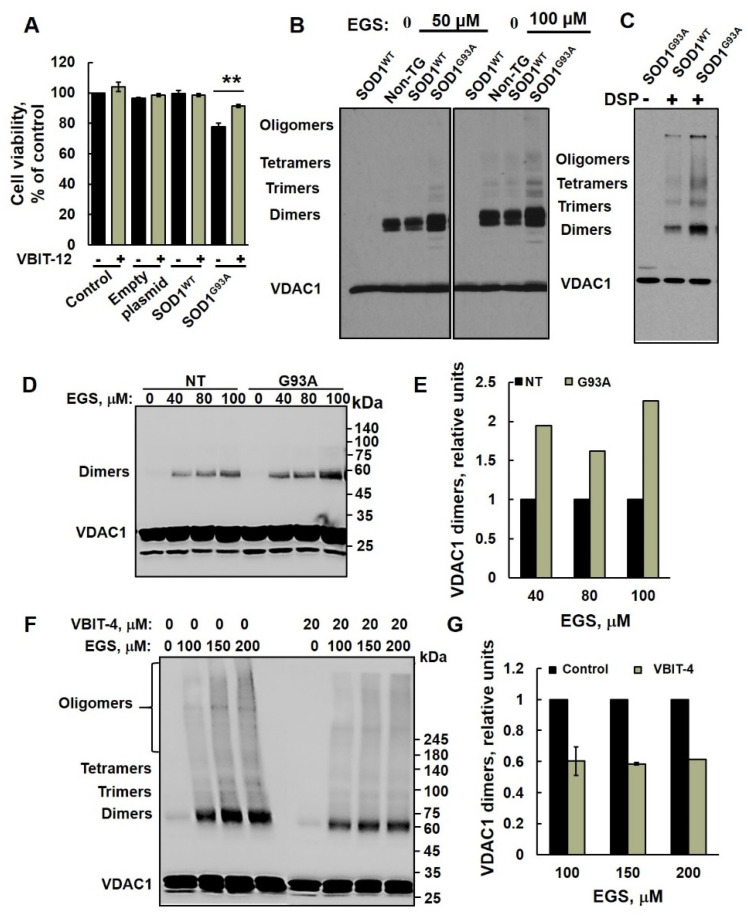
VDAC1 oligomerization is increased in mutant SOD1^G93A^ spinal cord mitochondria. (**A**) NSC-34 cells were transfected to express the human SOD1^WT^, the human mutant SOD1^G93A^, or non-transfected (control), and 24 h post-transfection, cells were incubated with or without VBIT-12 (15 µM) for 12 h. A cell viability analysis was performed using the CellTiter 96 AQueous One-Solution cell proliferation assay using a plate reader at 490 nm. (**B**,**C**) Spinal cord mitochondria (1 mg/mL) from non-transgenic, SOD1^WT^ or SOD1^G93A^ rats were incubated for 10 min at 30 °C in 10 mM Tricine, pH 8.2, with the indicated concentration of the crosslinking reagent EGS (**B**) or DSP (**C**). Sample buffer for electrophoresis containing 2-mercaptoethanol, except for DSP (S-S-containing reagent), was added, and the samples were subjected to SDS/PAGE (10% gel) and immunoblot analysis using anti-VDAC1 antibodies. (**D**,**E**) Spinal cord mitochondria (1 mg/mL, n = 3) from non-transgenic or SOD1^G93A^ mice were incubated for 20 min at 30 °C in PBS, pH 8.3, with the indicated concentration of EGS, and subjected to SDS-PAGE (10%), immunoblotting (**D**), and quantitative analysis (**E**). (**F**,**G**) Spinal cord mitochondria isolated from SOD1^G93A^ mice were pre-incubated with VBIT-4 (10 min, 20 μM) and subjected to cross linking as described in D. Samples were subjected to SDS/PAGE 4–15% gradient gel and immunoblotting using anti-VDAC1 antibodies (**F**) and to dimer quantitative analysis (**G**). VDAC1 dimer levels were quantified using ImageJ software. The positions of VDAC1 monomers, dimers, trimers, tetramers, and higher oligomers are indicated. The results are from triplicates of different biological repeats (n = 3) and are presented as the means ± SEM. ** *p* < 0.005.

VBIT-4 and VBIT-12 prevent both VDAC1 oligomerization and apoptotic cell death [49]. To test whether cell treatment with VBIT-12 can prevent mutant SOD1 toxicity in neurons, motor-neuron-like NSC-34 cells were transfected to express the human WT (SOD1^WT^) or mutant (SOD1^G93A^) SOD1 and treated with or without VBIT-12. Cell survival was monitored using the XTT assay. Whereas expressing mutant SOD1^G93A^ with no treatment reduced cell survival by ~25–30% compared to the survival of cells expressing SOD1^WT^, incubating these cells with VBIT-12 partially prevented the mutant SOD1 cell toxicity effect (Figure 3A).

### 2.3. VDAC1 Oligomerization Is Increased in Mutant SOD1^G93A^ Spinal Cord Mitochondria

Previously, we have demonstrated that apoptotic conditions lead to VDAC1 oligomerization, forming an oligomer with a large channel that enables release of pro-apoptotic proteins, such as cytochrome *c,* leading to cell death [21,26,51,52,53].

In order to determine whether the oligomeric state of VDAC1 is affected in ALS pathogenesis, we isolated spinal cord mitochondria from non-transgenic, transgenic SOD1^WT^, and mutant SOD1^G93A^ rats (Figure 3B,C) and spinal cord mitochondria from non-transgenic and mutant SOD1^G93A^ mice (Figure 3D–G) and performed chemical crosslinking with two different crosslinking reagents. Crosslinking of spinal cord mitochondrial proteins with EGS (Figure 3B) or DSP (a thiol-cleavable reagent, Figure 3C) resulted in the appearance of several bands interacting with anti-VDAC1 antibodies, corresponding to VDAC1 dimers, trimers, tetramers, and higher oligomeric states. Non-crosslinked VDAC1 monomers migrate as a 32 kDa protein band. Surprisingly, VDAC1 oligomeric levels were higher in the mutant SOD1^G93A^ spinal cord compared to SOD1^WT^ or non-transgenic rats (Figure 3B,C). Similarly, results were obtained with mitochondria isolated from SOD1^G93A^ mice (Figure 3D,E). As expected [49], VBIT-4 inhibited the oligomerization of VDAC1 in mutant SOD1^G93A^ spinal cord mitochondria (Figure 3F,G).

### 2.4. VDAC1 Expression Levels Are Increased in Mutant SOD1^G93A^ Spinal Motor Neurons

**Figure 4:**
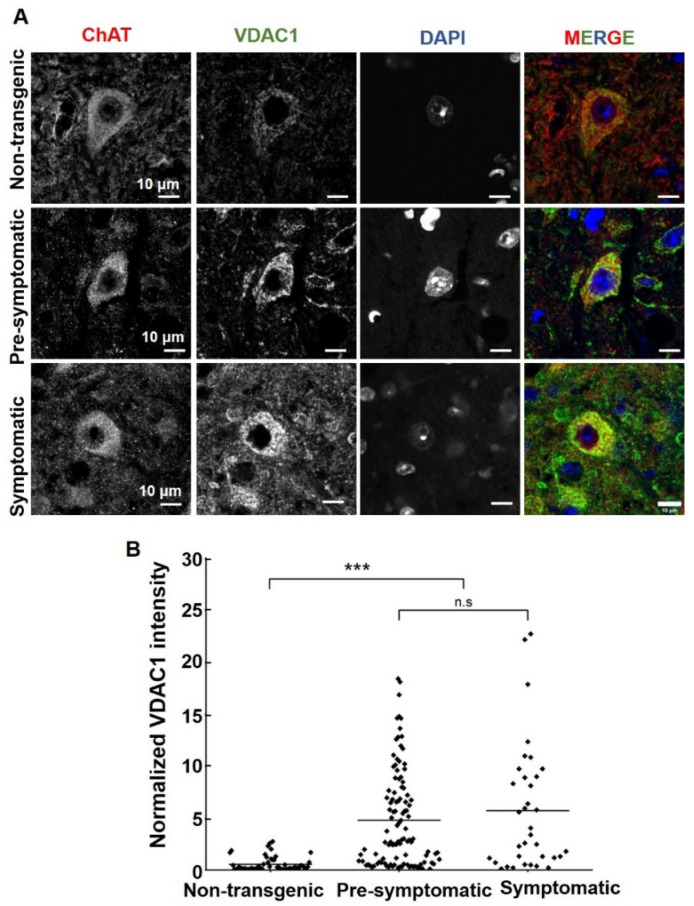
VDAC1 expression levels are increased in mutant SOD1^G93A^ spinal motor neurons. (**A**) Representative micrographs of paraformaldehyde-fixed paraffin-embedded lumbar spinal cord sections from non-transgenic, pre-symptomatic, and symptomatic mutant SOD1^G93A^ mice, co-immunofluorescence stained with the anti-ChAT antibody to detect motor neurons and anti-VDAC1. Cell nuclei were stained by DAPI. Scale bar = 10 μm. (**B**) Quantification of the relative fluorescence intensity of VDAC1 staining of lumbar spinal cord from non-transgenic, pre-symptomatic, and symptomatic mutant SOD1^G93A^ mice was calculated using NIS Element AR 5.21.03 software. About three different sections from three different mice of each group were analyzed. Statistics were calculated using OriginPro2022. Horizontal lines represent mean values for each group. *p*-values were determined using a Kruskal–Wallis test. *** *p* < 0.001; ns = non-significant.

VDAC1 expression levels in spinal cords derived from non-transgenic and mutant SOD1^G93A^ mice at pre-symptomatic and symptomatic stages were analyzed by immunofluorescence (IF) staining (Figure 4) and immunoblotting (Figure S2). VDAC1 levels were quantified in the ChAT-expressing motor neurons. The results demonstrate that VDAC1 levels are significantly higher in the spinal motor neurons of pre-symptomatic and symptomatic mutant SOD1^G93A^ mice compared to their non-transgenic littermates (Figure 4A,B). The levels of another mitochondrial protein, phosphatidylserine decarboxylase (PISD) [54], were found to be similar in the spinal motor neurons (stained with ChAT) of control and pre-symptomatic mice but were increased in the late symptomatic phase (Figure S1).

This suggests that the increase in VDAC1 expression in spinal motor neurons of pre-symptomatic mice is due to increased VDAC1 levels per mitochondria and not an increase in the total amount of mitochondria.

Similar VDAC1 levels were obtained in cervical or lumbar spinal cord extracts as analyzed using immunoblotting (Figure S2). This may result from analyzing VDAC1 in the whole tissue extract and not only in motor neurons, which represent a small percent of the spinal cord cells.

### 2.5. VBIT-12 Administration Significantly Improved Muscle endurance in Mutant SOD1^G93A^ Mice

Next, we tested whether VBIT-4 or VBIT-12 treatment of mutant SOD1^G93A^ transgenic mice had any protective effect on ALS disease progression. We previously demonstrated that VBIT-4 treatment via drinking water is well tolerated in mice [43,55,56]. Mutant SOD1^G93A^ female mice were divided into three groups (10 mice per group): a control group (DMSO), VBIT-4-, and VBIT-12-treated groups. Although treatment with VBIT-4 or VBIT-12 in the drinking water had no effect on disease onset (Figure S3A,B) or survival (Figure S3C–G), we were able to observe some benefit on muscle endurance as measured by grip strength in the hindlimbs of SOD1^G93A^ mice treated with VBIT-12 (Figure S3I).

**Figure 5:**
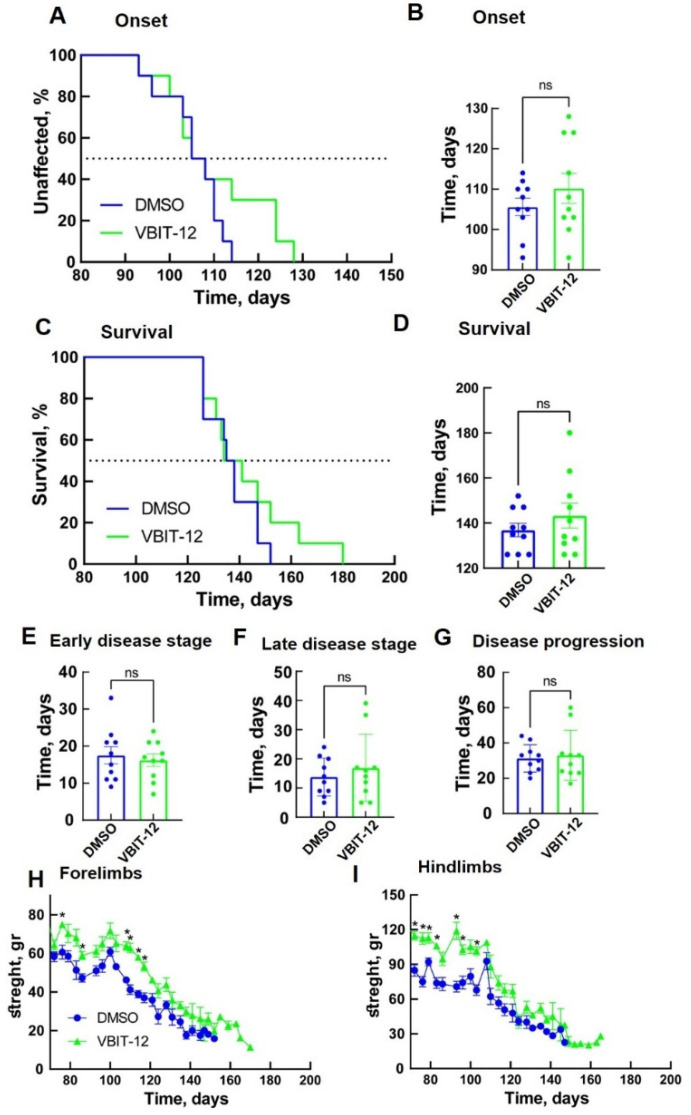
VBIT-12 administration by IP injection improved muscle endurance of SOD1^G93A^ mice but did not significantly extend disease onset and survival. Mean age of disease onset, defined as the time when mice reached peak body weight as a function of time (**A**,**B**), and disease end stage, defined as the time when the mouse could not right itself within 20 s when placed on its side (**C**,**D**) considering mutant SOD1^G93A^ mice IP-injected every second day with 200 µL DMSO 4% (control, *n* = 10, blue) or with VBIT-12 (26 mg/kg) (*n* = 10, green). The results are the means ± SD. (**E**–**G**) The means ± SD for the duration of early disease (from onset to 10% weight loss) (**E**), duration of late disease (from 10% weight loss to end stage) (**F**), and disease progression (from onset to end stage) (**G**) are presented. Plots of the averaged forelimb (**H**) and hindlimb (**I**) grip strength for SOD1^G93A^ female mice untreated (*n* = 10, blue) or treated with VBIT-12 (*n* = 10, green). SOD1^G93A^ mice were monitored up to the end stage. At each time point, the *p*-value was determined by Student’s *t*-test with SD. * *p* < 0.05; ns, non-significant.

Next, we selected VBIT-12, administrated intraperitoneally (IP), starting at 60 days. IP injection of VBIT-12 every other day showed a positive tendency in delaying disease onset (Figure 5A,B) and survival of SOD1^G93A^ mice, although this was not statistically significant (Figure 5C–G).

Examination of hindlimb and forelimb grip strength showed a rapid decline in DMSO-treated mice after disease onset (Figure 5H,I). However, SOD1^G93A^ mice subjected to IP injection of VBIT-12 maintained their limb muscle strength for a significantly more extended period of time. Overall, the VBIT-12-treated group showed a significantly greater pulling strength from their forelimbs (Figure 5H) and hindlimbs (Figure 5I) during disease progression compared to the control group. These results suggest that mice treated with VBIT-12 retain their muscular strength properties during the disease.

## 3. Discussion

The results presented in this study contribute to better understanding of the mechanism of misfolded SOD1-inducing toxicity in ALS pathogenesis. As we have shown in previous studies, misfolded SOD1 accumulation and mitochondrial association correlate with disease severity [3]. Misfolded SOD1 association with the OMM components leads to mitochondrial dysfunction and cellular toxicity by at least two different mechanisms: First, misfolded SOD1 interacts with VDAC1 at the OMM and affects its channel conductance for adenine nucleotides compromising the energy supply to the cells [31]. Second, misfolded SOD1 suppresses the import of mitochondrial proteins, thus, altering the mitochondrial protein composition [55].

Recently, we have shown that VDAC1 interacts directly with mutant SOD1 through the VDAC1-N-terminal domain [32]. In addition, we showed that different versions of N-terminal-derived peptides inhibited mutant SOD1 toxicity in NSC-34 neuronal cells and mESC-derived motor neurons [32]. Here, we identified for the first time the VDAC1 binding site in SOD1. Using a peptide array assay composed of hundreds of peptides derived from 19 VDAC1-interacting proteins including SOD1, we identified two SOD1-derived overlapping sequences which interacted with VDAC1. Importantly, these two peptides were detected by antibodies against a VDAC1 internal sequence, but not with antibodies recognizing the VDAC1 N-terminal domain. This finding suggests that the N-terminal domain of VDAC1 is bound to the SOD1-derived peptide (in the peptide array); thus, it is not available for the antibody’s recognition. Importantly, mutated SOD1 by binding to VDAC1 prevented its interaction with the SOD1-derived peptide in the array. Moreover, this interaction was validated by demonstrating that the VDAC1 interaction sequences in SOD1 produced as a synthetic peptide interact with purified VDAC1 with relatively high affinity.

The identified SOD1-derived peptide interacting with VDAC1 and the VDAC1-N-terminus-derived peptide interacting with SOD1 are possible candidates to target VDAC1-mutant SOD1 interactions, thereby, preventing its cytotoxicity. This is in line with recent progress in the development of small molecules, peptides, and antibodies that interfere with intracellular protein–protein interactions, having reached different clinical trials [56].

In addition to VDAC1-interacting SOD1-derived peptide, we also tested the effects of newly developed VDAC1-interacting molecules VBIT-4 and VBIT-12 on mutant SOD1-induced cell toxicity. These molecules prevent VDAC1 oligomerization, and, thereby, apoptosis and apoptosis-associated processes such as cytosolic Ca^2+^ elevation and ROS production with no effect on cells under physiological conditions [49]. VBIT-4 or VBIT-12 activity in preventing apoptosis and disease-associated processes was demonstrated in several disease models where VDAC1 is overexpressed, such as in type-2-diabetes (T2D) [43], lupus [44], and colitis [45]. Here, we demonstrated that in an ALS model, VDAC1 levels are significantly higher in spinal motor neurons from mutant SOD1^G93A^ mice, and VDAC1 oligomerization was increased in spinal cord mitochondria isolated from mutant SOD1^G93A^ mice and rats. Thus, we expected these molecules to attenuate the pathological conditions in a mouse model of ALS, namely, the mutant SOD1^G93A^ mice.

Here, we demonstrated that daily treatment of mutant SOD1^G93A^ transgenic mice with VBIT-12 significantly improved muscle function, without extending survival. A similar effect was previously shown by elevating PGC-1α activity, thus, sustaining mitochondrial biogenesis in the muscles of mutant SOD1 mice [57]. This may suggest that the VBIT-12 effect is due to preventing the mitochondrial dysfunction in the muscles, which cannot rescue the loss of motor axons or death of motor neurons. Thus, it is not able to slow the progression of the disease even though it improved muscle function. Therefore, VBIT-12, delivered systemically, could be used as a palliative treatment in ALS patients to preserve muscle function and improve daily physical activity.

VBIT-4, acting in some diseases as VBIT-12, was found to inhibit the release of mitochondrial DNA (mtDNA) via preventing VDAC1 oligomerization and inhibiting inflammation by activating cGAS/STING [45]. Interestingly, it has been shown recently that TDP-43 mutants triggered the release of mtDNA via the permeability transition pore (PTP) and induced inflammation by activating cGAS/STING [58]. Moreover, inhibiting VDAC1 oligomerization by VBIT-4 prevented the cytosolic accumulation of mtDNA and inflammation in iPSC-derived motor neurons from ALS patients carrying mutations in TDP-43 [58].

In conclusion, the identified VDAC1 binding site in SOD1, and the potential of this sequence as a cell-penetrating peptide to prevent mutant SOD1 cytotoxicity, provides an opportunity for the development of new ALS therapy. VBIT-12 treatment improving the muscle function of mutant SOD1 mice suggests that it may improve or preserve the daily functioning and quality of life of ALS patients.

## 4. Materials and Methods

### 4.1. Materials

Triton X-100 and Tween-20 were obtained from Sigma (St. Louis, MO, USA). Paraformaldehyde was obtained from Emsdiasum (Hatfield, PA, USA). Dulbecco’s modified Eagle’s medium (DMEM) was obtained from Gibco (Grand Island, NY, USA). Normal goat serum (NGS) and the supplements fetal bovine serum (FBS), L-glutamine, and penicillin-streptomycin were obtained from Biological Industries (Beit Haemek, Israel). Primary and secondary antibodies, their source, and dilutions are detailed in Table S1. XTT (2,3-bis-(2-methoxy-4-nitro-5-sulfophenyl)-2H-tetrazolium-5-carboxanilide) was obtained from Promega. Peptide arrays (BGUN_SB-002) were produced by INTAVIS Peptide Services (GmbH & Co. KG, Tübingen, Germany), composing 768 peptide sequences derived from 19 VDAC1-interacting proteins.

### 4.2. Synthetic Peptides

Customized peptides, SOD1-derived peptide (GPVKVWGSIKGLTEGLHGFHVHEFG) (96.02% purity) or VDAC1-N-terminus-derived peptide (N-Ter Δ(21-26)-Antp (1-MAVPPTYADLGKSARDVFTK-20-RQIKIWFQNRRMKWKK)) (95% purity) were synthesized by GL Biochem (Shanghai, China).

### 4.3. Peptide Arrays

Customized 768-peptide sequences derived from 19 VDAC1-interacting proteins produced by INTAVIS Peptide Services (GmbH & Co. KG, Tübingen, Germany) were arrayed on a glass slide. The interaction of purified VDAC1 with glass-bound peptide arrays (BGUN_SB-002) was assessed by slide washing with TBST buffer (150 mM NaCl, 50 mM Tris/HCl, pH 7.4, and 0.1% Tween-20), followed by overnight incubation with blocking buffer (Tris-buffered saline, pH 7.8, containing 2.5% low-fat milk, *w*/*v*) at 4 °C. Next, the peptide array glass slides were incubated with purified VDAC1 (64 nM) in 400 µL of protein-interacting buffer (20 mM HEPES, 100 mM KCl, pH,7.4, PBS) at room temperature for 4 h in a humidified chamber. Then, the peptide array glass slides were washed (three times, 10 min each) with TBST and incubated overnight at 4 °C with anti-VDAC1 antibody against an internal sequence (ab15895) or against the N-terminus (ab154856) (1:5000). The peptide array glass slides were washed with 0.1% Tween-20 in TBS buffer/Tris-buffered saline (150 mM NaCl, 50 mM Tris/HCl, pH 7.4 TBST), followed by incubation for 2 h at RT with HRP-conjugated anti-mouse or anti-rabbit (1:10,000) or anti-goat (1:20,000) IgG as a secondary antibody. Enhanced chemiluminescent substrate (Pierce Chemical, Rockford, IL, USA) was used to detect HRP. The signal was captured in the dark using FUSION-FX (Vilber Lourmat) software.

### 4.4. VDAC1-Based Peptide Interaction Analysis Using Microscale Thermophoresis (MST)

Purified VDAC1 was fluorescently labeled using a NanoTemper blue protein-labeling kit. Fluorescently-labeled VDAC1 (138 nM) was incubated with different concentrations of peptides (1–30 μM) in 10mM Tricine buffer containing 100mM NaCl. After 20 min of incubation, 3–5-μL aliquots were loaded into MST-grade glass capillaries (NanoTemper Technologies, Munich, Germany), and thermophoresis was measured with a NanoTemper Monolith-NT115 system (20/40% light-emitting diode, 40% IR laser power).

### 4.5. Cell Culture

SH-SY5Y and NSC-34 cells were grown at 37 °C and with 5% CO_2_ in Dulbecco’s modified Eagle medium (DMEM) supplemented with 10% tetracycline-free fetal bovine serum (FBS), L-Glutamine (2 mM), and penicillin (100 U/mL)/streptomycin (0.1 mg/mL). Transfection was performed by using TurboFect (Thermo, Waltham, MA, USA) according to the manufacturer’s protocol. When co-transfections were performed, empty plasmids were transfected as controls. According to the manufacturer’s protocol, cell viability was analyzed using the CellTiter 96 AQueous one-solution cell proliferation assay (Promega) and plate reader at 490 nm.

### 4.6. Crosslinking Experiments

Rat spinal cord mitochondria (1 mg/mL) from non-transgenic, SOD1^WT^ or SOD1^G93A^ were incubated with 50 and 100 µM of EGS and 400 µM of DSP crosslinkers for 10 min at 30 °C in a solution containing 10 mM Tricine, at pH 8.2. Both crosslinkers were dissolved in DMSO immediately before use. The DMSO concentration in the control and reagent-containing samples was up to 2% (*v*/*v*). Samples treated with the crosslinking reagent DSP were treated with SDS/PAGE sample buffer, lacking 2-mercaptoethanol.

### 4.7. Purified Proteins

The VDAC1 protein was purified from rat liver mitochondria, as previously described [22]. Briefly, rat liver mitochondria (5 mg/mL) in 10 mM Tris-HCl, pH 7.2, were incubated with 2% LDAO on ice for 20 min, followed by centrifugation (30 min, 14,000× *g*), and the obtained supernatant was loaded onto a dry celite:hydroxyapatite (2:1) column. VDAC1 was eluted with a solution containing 2% LDAO, 10 mM Tris-HCl, pH 7.2, 50 mM NaCl, and 22 mM NaH_2_PO_4_. The VDAC1-containing fractions were dialyzed against 10 mM Tris-HCl, pH 7.2, and subjected to a second chromatography step on a carboxymethyl-cellulose (CMC) column from which VDAC1 was eluted with a solution containing 10 mM Tris-HCl, pH 7.2, 0.3% LDAO, and 500 mM NaCl. The VDAC1-containing fractions were collected and used for VDAC1 channel conductance and microscale thermophoresis (MST) assays.

Recombinant SOD1^WT^, SOD1^G93A^, and SOD1^G85R^ were purified as previously described [59]. Briefly, the proteins were expressed as 6His-tagged (N-term) soluble proteins in BL21 cells, and the expression of the recombinant proteins was induced by addition of 0.5 mM IPTG, following purification using a 5mL HisTrap FF column (GE Healthcare, Chicago, IL, USA). The peak fractions were dialyzed overnight at 4 °C against a storage buffer (50 mM Na^+^/phosphate, pH 7.6, 0.1 M NaCl, and 10% glycerol), concentrated by ultrafiltration (10 kDa cutoff, Millipore, USA), centrifuged at 110,000× *g* at 4 °C for 1 h using ultracentrifuge (Sorvall M120 Discovery, Waltham, MA, USA), and stored at −20 °C until use. Protein concentration was measured with the Bradford method using bovine serum albumin as the standard.

### 4.8. Gel Electrophoresis and Immunoblotting

For immunoblotting, samples were subjected to gel electrophoresis and immunoblotting using the primary antibodies, and then with HRP-conjugated anti-mouse or anti-rabbit IgG (sources and dilutions are detailed in Table S1) followed by detection of HRP activity. Band intensities were analyzed by densitometry using FUSION-FX (Vilber Lourmat, Collégien, France) software.

### 4.9. Animals and VBIT-4 or VBIT-12 Treatment

Mutant SOD1^G93A^ transgenic mice (B6SJL-TgN-SOD1-G93A-1Gur) were obtained from The Jackson Laboratory (Bar Harbor, ME, USA) for the experiments. All experiments were approved by the Animal Care and Use Committee of Ben-Gurion University of the Negev, as required by Israeli legislation, and all efforts were taken to minimize animal suffering.

VBIT-4 and VBIT-12 were dissolved in DMSO (80 mg/mL) and then diluted in drinking water while being added in a dropwise manner into the acidic water to a final concentration of 0.0625 mg/mL. Considering an estimated daily water consumption of a 25 g mouse as 6–8 mL per mouse, the daily dose of VBIT-4 and VBIT-12 was determined. Assuming a mouse drinks about 8 mL of water daily, it consumed 20 mg/kg of the compound. Control untreated WT mice received water containing DMSO (0.36%) at the final concentration as in VBIT-4 or VBIT-12 drinking solutions. Mutant SOD1^G93A^ female mice were randomly divided to receive drinking water containing VBIT-4, VBIT-12, or DMSO for the control. Water consumption was verified daily.

For intraperitoneal (IP) injection, VBIT-12 (80 mg/mL) in DMSO was diluted with saline, pH 7.0, to a final concentration of 3.25 mg/mL. Mice received 200 µL by IP injection resulting in a final VBIT-12 concentration of ~26 mg/Kg. VBIT-12 was IP injected every second day starting from day 60 for 12 weeks, and then the VBIT-12 final concentration was reduced to ~13 mg/Kg. The control group was IP injected with around 200 µL of DMSO (4%).

### 4.10. Immunofluorescence (IF) Staining

Immunofluorescence staining was performed on formalin-fixed and paraffin-embedded spinal cord sections (5 µm). Sections were deparaffinized by placing the slides at 60 °C for 1 h and using xylene, followed by rehydration with a graded ethanol series (100%–50%). Antigen retrieval was performed in 0.01 M citrate buffer (pH 6.0) at 95–98 °C for 20 min. After washing sections in PBS, pH 7.4, sections were incubated in 10% normal goat serum for 2 h, followed by overnight incubation at 4 °C with primary antibodies (Table S1). Sections were washed thoroughly with PBST and incubated with the fluorescently labeled secondary antibodies (Table S1) for 2 h at RT in the dark. Following a wash with PBS, coverslips were incubated with DAPI (0.5µ g/mL) for 15 min in the dark, and carefully washed, dried, and mounted on slides with fluoroshield (ImmunoBioSciences, Mukilteo, WA, USA) mounting medium. After overnight drying at 4 °C, images were acquired using a NIKON C2Plus laser unit docked to a Nikon Eclipse *Ti confocal* microscope using a 60× oil immersion objective. Scanning settings for individual channels were kept constant across the samples, and final image properties (intensity, brightness, contrast) were adjusted using the same settings for all images. VDAC1 fluorescence intensity was quantified in ChAT positive cells from spinal cord sections of three different mice from each group (NT, PRE, SYMP) using NIS Element AR 5.21.03 software. VDAC1 fluorescence intensity in each cell was divided by the area of interest, and nuclei were excluded. Finally, the level of fluorescence intensity in each group was normalized to the control.

### 4.11. Immunoprecipitation

Purified VDAC1 protein (2 µg) was first incubated with increasing concentrations of SOD1-derived peptide (0, 5, 15, and 30 µg) for 1 h at 37 °C. An additional 30 min incubation time was used also in the presence of purified SOD1^G93A^ (2 µg). After incubation, the purified proteins combined with SOD1-derived peptides were solubilized in cold immunoprecipitation (IP) buffer (50 mM Tris-HCl (pH 7.4) and 150 mM NaCl) and incubated overnight at 4 °C, while rotating, with anti-SOD1 antibody (Santa-Cruz Biotechnology, Dallas, TX, USA), previously crosslinked to Dynabeads™ protein G (Thermo) and diluted in PBS with 0.02% Tween-20 according to the manufacturer’s protocol. Following magnetic separation, unbound samples were boiled for 5 min with sample buffer and withdrawn for immunoblotting analysis. The beads were magnetically isolated and washed three times with IP buffer. Bound samples were eluted by boiling in 2× sample buffer for 5 min and withdrawn for immunoblotting analysis.

### 4.12. Quantification and Statistical Analyses

All data used for statistical analyses indicated in the legends to figures were acquired from at least three biological replicates. Data were collected throughout the study and analyzed using RStudio Team (2020) version 1.3.959. (RStudio: Integrated Development for R. RStudio, PBC, Boston, MA, USA URL http://www.rstud io.com/). Kaplan–Meier curves were generated with the survival package v3.2-3 and plotted with the survminer package v0.4.7. Bar and line plots were generated with the ggplot2 package v3.3.2. Statistical analysis was further performed with stats v3.6.3 (R Core Team) and other packages loaded into the RStudio environment: dplyr v1.0.0, tidyverse v1.3.0., and multicomp v1.4–13. Multi-comparison of the means was completed using a one-way ANOVA with Tukey posthoc analysis, with a p value of <0.05 statistically significant (*** < 0.001; ** < 0.01; * < 0.05) loaded in the multicomp RStudio.

## Data Availability

Not applicable.

## Supplementary Materials

The following supporting information can be downloaded at: https://www.mdpi.com/article/10.3390/ijms23179946/s1.

## References

[1] Da Cruz S., Cleveland D.W. (2011). Understanding the role of TDP-43 and FUS/TLS in ALS and beyond. Curr. Opin. Neurobiol..

[2] Kaur S.J., McKeown S.R., Rashid S. (2016). Mutant SOD1 mediated pathogenesis of Amyotrophic Lateral Sclerosis. Gene.

[3] Abu-Hamad S., Kahn J., Leyton-Jaimes M.F., Rosenblatt J., Israelson A. (2017). Misfolded SOD1 Accumulation and Mitochondrial Association Contribute to the Selective Vulnerability of Motor Neurons in Familial ALS: Correlation to Human Disease. ACS Chem. Neurosci..

[4] Israelson A., Ditsworth D., Sun S., Song S., Liang J., Hruska-Plochan M., McAlonis-Downes M., Abu-Hamad S., Zoltsman G., Shani T. (2015). Macrophage Migration Inhibitory Factor as a Chaperone Inhibiting Accumulation of Misfolded SOD1. Neuron.

[5] Kiskinis E., Sandoe J., Williams L.A., Boulting G.L., Moccia R., Wainger B.J., Han S., Peng T., Thams S., Mikkilineni S. (2016). Pathways Disrupted in Human ALS Motor Neurons Identified through Genetic Correction of Mutant. Cell Stem Cell.

[6] Saxena S., Cabuy E., Caroni P. (2009). A role for motoneuron subtype-selective ER stress in disease manifestations of FALS mice. Nat. Neurosci..

[7] Urushitani M., Kurisu J., Tsukita K., Takahashi R. (2002). Proteasomal inhibition by misfolded mutant superoxide dismutase 1 induces selective motor neuron death in familial amyotrophic lateral sclerosis. J. Neurochem..

[8] Kabashi E., Agar J.N., Strong M., Durham H.D. (2012). Impaired proteasome function in sporadic amyotrophic lateral sclerosis. Amyotroph. Lateral Scler..

[9] Tokuda E., Brännström T., Andersen P.M., Marklund S.L. (2016). Low autophagy capacity implicated in motor system vulnerability to mutant superoxide dismutase. Acta Neuropathol. Commun..

[10] Leal S.S., Cardoso I., Valentine J.S., Gomes C.M. (2013). Calcium Ions Promote Superoxide Dismutase 1 (SOD1) Aggregation into Non-fibrillar Amyloid. J. Biol. Chem..

[11] Barber S.C., Mead R., Shaw P.J. (2006). Oxidative stress in ALS: A mechanism of neurodegeneration and a therapeutic target. Biochim. Biophys. Acta (BBA)—Mol. Basis Dis..

[12] van den Bosch L., van Damme P., Bogaert E., Robberecht W. (2006). The role of excitotoxicity in the pathogenesis of amyotrophic lateral sclerosis. Biochim. Biophys. Acta (BBA)—Mol. Basis Dis..

[13] Beretta S., Sala G., Mattavelli L., Ceresa C., Casciati A., Ferri A., Carrì M.T., Ferrarese C. (2003). Mitochondrial dysfunction due to mutant copper/zinc superoxide dismutase associated with amyotrophic lateral sclerosis is reversed by N-acetylcysteine. Neurobiol. Dis..

[14] Velde C.V., Miller T.M., Cashman N.R., Cleveland D.W. (2008). Selective association of misfolded ALS-linked mutant SOD1 with the cytoplasmic face of mitochondria. Proc. Natl. Acad. Sci. USA.

[15] Halestrap A.P., Doran E., Gillespie J.P., O’Toole A. (2000). Mitochondria and cell death. Biochem. Soc. Trans..

[16] Wallace D.C. (2005). A mitochondrial paradigm of metabolic and degenerative diseases, aging, and cancer: A dawn for evolutionary medicine. Annu. Rev. Genet..

[17] Bannwarth S., Ait-El-Mkadem S., Chaussenot A., Genin E.C., Lacas-Gervais S., Fragaki K., Berg-Alonso L., Kageyama Y., Serre V., Moore D.G. (2014). A mitochondrial origin for frontotemporal dementia and amyotrophic lateral sclerosis through CHCHD10 involvement. Brain.

[18] Darshi M., Mendiola V.L., Mackey M.R., Murphy A.N., Koller A., Perkins G.A., Ellisman M.H., Taylor S.S. (2011). ChChd3, an inner mitochondrial membrane protein, is essential for maintaining crista integrity and mitochondrial function. J. Biol. Chem..

[19] Kholmukhamedov E.L., Czerny C., Lovelace G., Beeson K.C., Baker T., Johnson C.B., Pediaditakis P., Teplova V.V., Tikunov A., MacDonald J. (2010). The role of the voltage-dependent anion channels in the outer membrane of mitochondria in the regulation of cellular metabolism. Biofizika.

[20] Shoshan-Barmatz V., de Pinto V., Zweckstetter M., Raviv Z., Keinan N., Arbel N. (2010). VDAC, a multi-functional mitochondrial protein regulating cell life and death. Mol. Asp. Med..

[21] Shoshan-Barmatz V., Ben-Hail D. (2012). VDAC, a multi-functional mitochondrial protein as a pharmacological target. Mitochondrion.

[22] Shoshan-Barmatz V., Ben-Hail D., Admoni L., Krelin Y., Tripathi S.S. (2015). The mitochondrial voltage-dependent anion channel 1 in tumor cells. Biochim. Biophys. Acta.

[23] Raghavan A., Sheiko T., Graham B.H., Craigen W.J. (2012). Voltage-dependant anion channels: Novel insights into isoform function through genetic models. Biochim. Biophys. Acta.

[24] de Pinto V., Guarino F., Guarnera A., Messina A., Reina S., Tomasello F.M., Palermo V., Mazzoni C. (2010). Characterization of human VDAC isoforms: A peculiar function for VDAC3?. Biochim. Biophys. Acta.

[25] Shoshan-Barmatz V., Maldonado E.N., Krelin Y. (2017). VDAC1 at the crossroads of cell metabolism, apoptosis and cell stress. Cell Stress.

[26] Shoshan-Barmatz V., Mizrachi D., Keinan N. (2013). Oligomerization of the mitochondrial protein VDAC1: From structure to function and cancer therapy. Prog. Mol. Biol. Transl. Sci..

[27] Hanahan D., Weinberg R.A. (2011). Hallmarks of cancer: The next generation. Cell.

[28] Shoshan-Barmatz V., Israelson A., Brdiczka D., Sheu S.S. (2006). The Voltage-Dependent Anion Channel (VDAC): Function in Intracellular Signalling, Cell Life and Cell Death. Curr. Pharm. Des..

[29] Shoshan-Barmatz V., Golan M. (2012). Mitochondrial VDAC1: Function in cell life and death and a target for cancer therapy. Curr. Med. Chem..

[30] Shoshan-Barmatz V., Mizrachi D. (2012). VDAC1: From structure to cancer therapy. Front. Oncol..

[31] Israelson A., Arbel N., da Cruz S., Ilieva H., Yamanaka K., Shoshan-Barmatz V., Cleveland D.W. (2010). Misfolded Mutant SOD1 Directly Inhibits VDAC1 Conductance in a Mouse Model of Inherited ALS. Neuron.

[32] Shteinfer-Kuzmine A., Argueti S., Gupta R., Shvil N., Abu-Hamad S., Gropper Y., Hoeber J., Magrì A., Messina A., Kozlova E.N. (2019). A VDAC1-Derived N-Terminal Peptide Inhibits Mutant SOD1-VDAC1 Interactions and Toxicity in the SOD1 Model of ALS. Front. Cell Neurosci..

[33] Pittala M.G.G., Reina S., Cubisino S.A.M., Cucina A., Formicola B., Cunsolo V., Foti S., Saletti R., Messina A. (2020). Post-Translational Modification Analysis of VDAC1 in ALS-SOD1 Model Cells Reveals Specific Asparagine and Glutamine Deamidation. Antioxidants.

[34] Shoshan-Barmatz V., Shteinfer-Kuzmine A., Verma A. (2020). VDAC1 at the Intersection of Cell Metabolism, Apoptosis, and Diseases. Biomolecules.

[35] Arif T., Krelin Y., Nakdimon I., Benharroch D., Paul A., Dadon-Klein D., Shoshan-Barmatz V. (2017). VDAC1 is a molecular target in glioblastoma, with its depletion leading to reprogrammed metabolism and reversed oncogenic properties. Neuro Oncol..

[36] Shoshan-Barmatz V., Krelin Y., Shteinfer-Kuzmine A., Arif T. (2017). Voltage-Dependent Anion Channel 1 As an Emerging Drug Target for Novel Anti-Cancer Therapeutics. Front. Oncol..

[37] Arif T., Vasilkovsky L., Refaely Y., Konson A., Shoshan-Barmatz V. (2014). Silencing VDAC1 Expression by siRNA Inhibits Cancer Cell Proliferation and Tumor Growth In Vivo, Molecular therapy. Nucleic Acids.

[38] Manczak M., Reddy P.H. (2012). Abnormal interaction of VDAC1 with amyloid beta and phosphorylated tau causes mitochondrial dysfunction in Alzheimer’s disease. Hum. Mol. Genet..

[39] Cuadrado-Tejedor M., Vilarino M., Cabodevilla F., del Rio J., Frechilla D., Perez-Mediavilla A. (2011). Enhanced expression of the voltage-dependent anion channel 1 (VDAC1) in Alzheimer’s disease transgenic mice: An insight into the pathogenic effects of amyloid-beta. J. Alzheimer’s Dis. JAD.

[40] Perez-Gracia E., Torrejon-Escribano B., Ferrer I. (2008). Dystrophic neurites of senile plaques in Alzheimer’s disease are deficient in cytochrome c oxidase. Acta Neuropathol..

[41] Ahmed M., Muhammed S.J., Kessler B., Salehi A. (2010). Mitochondrial proteome analysis reveals altered expression of voltage dependent anion channels in pancreatic beta-cells exposed to high glucose. Islets.

[42] Gong D., Chen X., Middleditch M., Huang L., Amarsingh G.V., Reddy S., Lu J., Zhang S., Ruggiero K., Phillips A.R. (2009). Quantitative proteomic profiling identifies new renal targets of copper(II)-selective chelation in the reversal of diabetic nephropathy in rats. Proteomics.

[43] Zhang E., Mohammed Al-Amily I., Mohammed S., Luan C., Asplund O., Ahmed M., Ye Y., Ben-Hail D., Soni A., Vishnu N. (2019). Preserving Insulin Secretion in Diabetes by Inhibiting VDAC1 Overexpression and Surface Translocation in β Cells. Cell Metab..

[44] Kim J., Gupta R., Blanco L.P., Yang S., Shteinfer-Kuzmine A., Wang K., Zhu J., Kang K., Zhu X., Park S.-J. (2019). VDAC oligomers form mitochondrial pores to release mtDNA fragments and promote lupus-like disease. Science.

[45] Verma A., Pittala S., Alhozeel B., Shteinfer-Kuzmine A., Ohana E., Gupta R., Chung J.H., Shoshan-Barmatz V. (2021). The role of the mitochondrial protein VDAC1 in inflammatory bowel disease: A potential therapeutic target. Mol. Ther..

[46] Abu-Hamad S., Zaid H., Israelson A., Nahon E., Shoshan-Barmatz V. (2008). Hexokinase-I protection against apoptotic cell death is mediated via interaction with the voltage-dependent anion channel-1: Mapping the site of binding. J. Biol. Chem..

[47] Smilansky A., Dangoor L., Nakdimon I., Ben-Hail D., Mizrachi D., Shoshan-Barmatz V. (2015). The Voltage-dependent Anion Channel 1 Mediates Amyloid beta Toxicity and Represents a Potential Target for Alzheimer Disease Therapy. J. Biol. Chem..

[48] Zaid H., Abu-Hamad S., Israelson A., Nathan I., Shoshan-Barmatz V. (2005). The voltage-dependent anion channel-1 modulates apoptotic cell death. Cell Death Differ..

[49] Ben-Hail D., Begas-Shvartz R., Shalev M., Shteinfer-Kuzmine A., Gruzman A., Reina S., de Pinto V., Shoshan-Barmatz V. (2016). Novel Compounds Targeting the Mitochondrial Protein VDAC1 Inhibit Apoptosis and Protect against Mitochondrial Dysfunction. J. Biol. Chem..

[50] Pettersen E.F., Goddard T.D., Huang C.C., Couch G.S., Greenblatt D.M., Meng E.C., Ferrin T.E. (2004). UCSF Chimera--a visualization system for exploratory research and analysis. J. Comput. Chem..

[51] Zalk R., Israelson A., Garty E.S., Azoulay-Zohar H., Shoshan-Barmatz V. (2005). Oligomeric states of the voltage-dependent anion channel and cytochrome c release from mitochondria. Biochem. J..

[52] Keinan N., Tyomkin D., Shoshan-Barmatz V. (2010). Oligomerization of the mitochondrial protein voltage-dependent anion channel is coupled to the induction of apoptosis. Mol. Cell. Biol..

[53] Keinan N., Pahima H., Ben-Hail D., Shoshan-Barmatz V. (2013). The role of calcium in VDAC1 oligomerization and mitochondria-mediated apoptosis. Biochim. Biophys. Acta.

[54] di Bartolomeo F., Wagner A., Daum G. (2017). Cell biology, physiology and enzymology of phosphatidylserine decarboxylase. Biochim. Biophys. Acta Mol. Cell Biol. Lipids.

[55] Li Q., Velde C.V., Israelson A., Xie J., Bailey A.O., Dong M.Q., Chun S.J., Roy T., Winer L., Yates J.R. (2010). ALS-linked mutant superoxide dismutase 1 (SOD1) alters mitochondrial protein composition and decreases protein import. Proc. Natl. Acad. Sci. USA.

[56] Lu H., Zhou Q., He J., Jiang Z., Peng C., Tong R., Shi J. (2020). Recent advances in the development of protein-protein interactions modulators: Mechanisms and clinical trials. Signal Transduct. Target. Ther..

[57] da Cruz S., Parone P.A., Lopes V.S., Lillo C., McAlonis-Downes M., Lee S.K., Vetto A.P., Petrosyan S., Marsala M., Murphy A.N. (2012). Elevated PGC-1alpha activity sustains mitochondrial biogenesis and muscle function without extending survival in a mouse model of inherited ALS. Cell Metab..

[58] Yu C.H., Davidson S., Harapas C.R., Hilton J.B., Mlodzianoski M.J., Laohamonthonkul P., Louis C., Low R.R.J., Moecking J., de Nardo D. (2020). TDP-43 Triggers Mitochondrial DNA Release via mPTP to Activate cGAS/STING in ALS. Cell.

[59] Shvil N., Banerjee V., Zoltsman G., Shani T., Kahn J., Abu-Hamad S., Papo N., Engel S., Bernhagen J., Israelson A. (2018). MIF inhibits the formation and toxicity of misfolded SOD1 amyloid aggregates: Implications for familial ALS. Cell Death Dis..

