# Supplementary material for: Targeting the Mitochondrial Protein VDAC1 as a Potential Therapeutic Strategy in ALS
Source: Int J Mol Sci. 2022 Sep 1;23(17):9946. doi: 10.3390/ijms23179946 (PMC9456491; doi:10.3390/ijms23179946)
Supplement: Supplementary file 1 [file ijms-23-09946-s001.zip › ijms-1766707-supplementary.pdf]

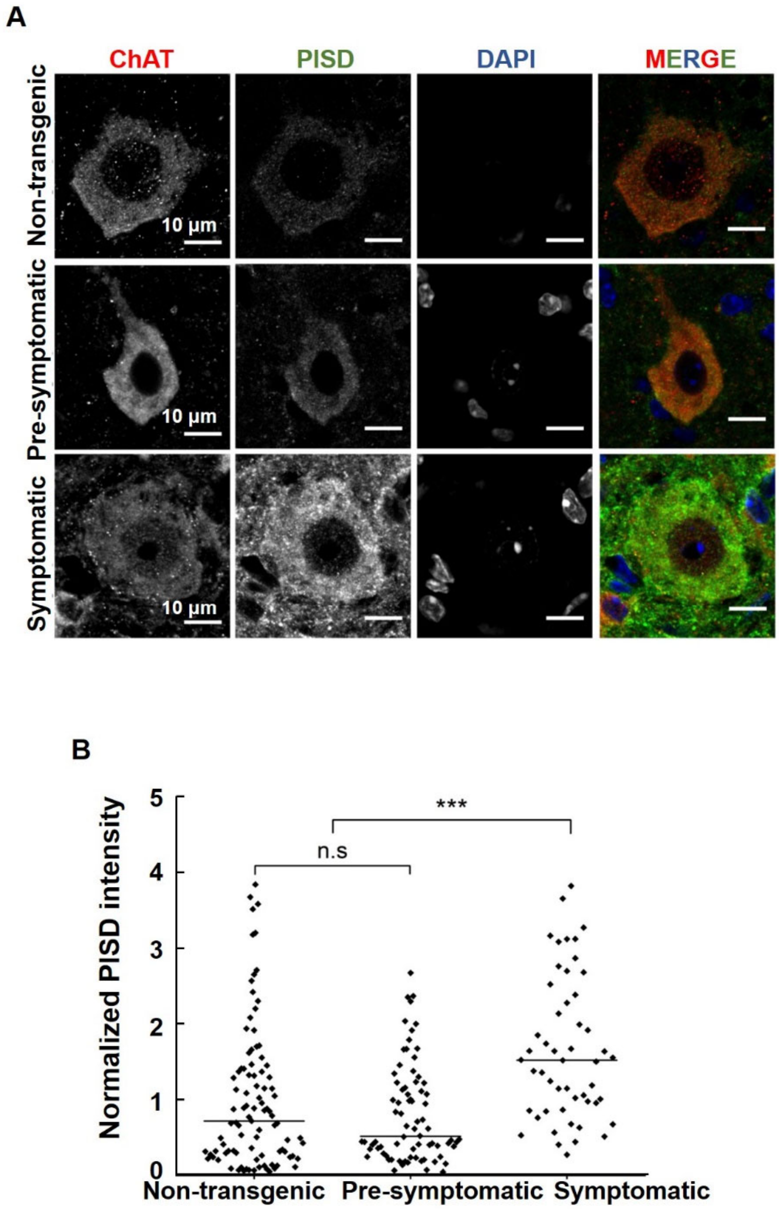

Figure S1. PISD expression levels are increased only at the symptomatic stage in mutant SOD1^{G93A} spinal motor neurons. (**A**) Representative micrographs of paraformaldehyde-fixed paraffin-embedded lumbar spinal cord sections from non-transgenic, pre-symptomatic and symptomatic mutant SOD1^{G93A} mice, co-immunofluorescence stained with the anti-ChAT antibody to detect motor neurons and anti-PISD as a mitochondrial control protein. Cell nuclei were stained by DAPI. Scale bar=10 µm. (**B**) Quantification of the relative fluorescence intensity of PISD staining of lumbar spinal cord from non-transgenic, pre-symptomatic, and symptomatic mutant SOD1^{G93A} mice was calculated using NIS Element AR 5.21.03 software. About three different sections from three different mice of each group were analysed. Statistics were calculated by OriginPro2022. Horizontal lines represent median values for each group. p-values were determined by a Kruskal–Wallis test. ***p<0.001, ns= non-significant.

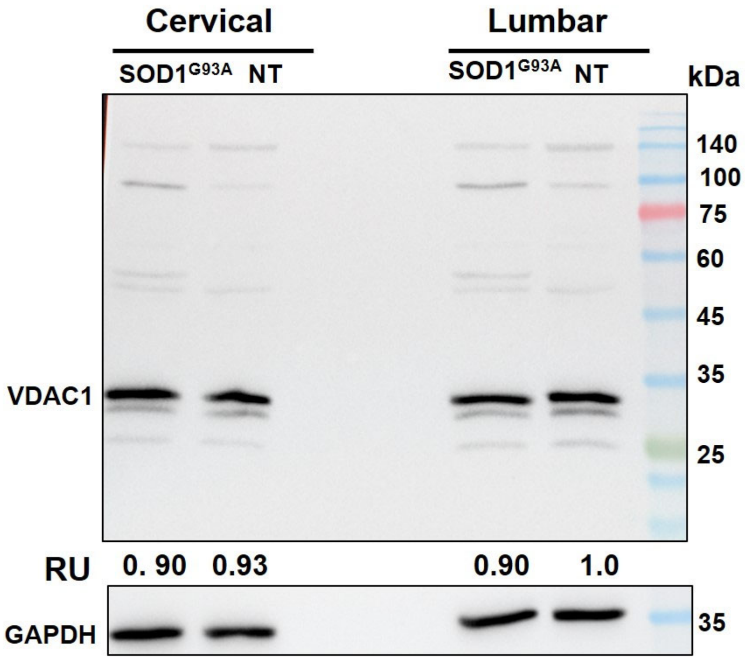

Figure S2. VDAC1 protein expression in the cervical and lumbar spinal-cord extracts from non-transgenic and mutant SOD1^{G93A} mice. The level of VDAC1 protein was assessed by immunoblotting in the cervical and lumbar spinal-cord extracts of transgenic mice expressing mutant SOD1^{G93A} or in age-matched non-transgenic control mice. GAPDH levels were detected for a loading control.

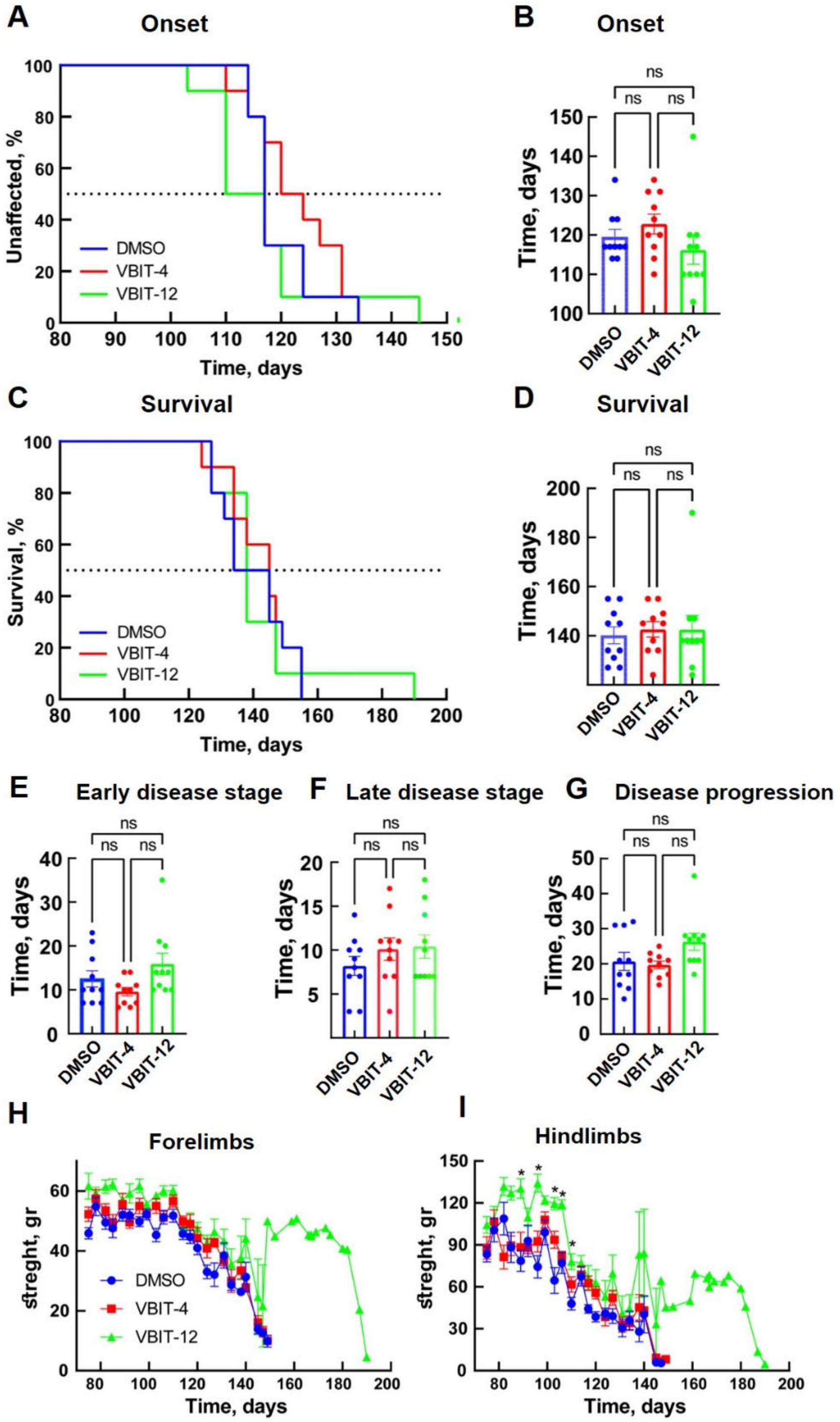

Figure S3. VBIT-4 and VBIT-12 administration in drinking water improved muscle endurance of mutant SOD1^{G93A} mice, but had no effect on disease onset and survival.

Mean age of disease onset, defined as the time when mice reached peak body weight **(A, B)**, and disease end stage, defined as the time when the mouse could not right itself within 20 s when placed on its side (**C, D**) of mutant SOD1^{G93A} mice without (control, *n* = 10, blue) and with treatment of VBIT-4 (*n* = 10, red) or VBIT-12 (*n* = 10, green) through drinking water. The results are the means ± SD. (**E–G**) Mean (± SD) duration of early disease (from onset to 10% weight loss) (**E**), duration of late disease (from 10% weight loss to end stage) (**F**), and disease progression (from onset to end stage) (**G**). At each time point, the *p*-value was determined by a Student's t-test. Error bars denote SDs. Plot of averaged forelimb (**H**) and hindlimb (**I**) grip strength for SOD1^{G93A} female mice untreated (*n* = 10, blue), treated with VBIT-4 (*n* = 10, red), or treated with VBIT-12 (*n* = 10, green) through the drinking water. SOD1^{G93A} mice were monitored up to the end stage. The untreated SOD1^{G93A} mice received water with the same concentration of DMSO as controls. At each time point, the *p*-value was determined by a Student's t-test. Error bars denote SDs.

Table S1. Antibodies used in the study

**Antibody**	**Source and Cat. No.**	**WB**	**IF**
ChAT	Gentex, GTX113164		1:500
Mouse monoclonal anti-GAPDH	Abcam, Cambridge, UK, ab9484	1:2000	-
Mouse monoclonal anti-PISD	Santa Cruz Biotechnology, Texas USA, sc-390070		1:100
SOD1	Santa Cruz Biotechnology, Texas USA, SC-101523		
Rabbit polyclonal anti-VDAC1	Abcam, Cambridge, UK, ab15895	1:5000	
Mouse monoclonal anti-VDAC1	Abcam, Cambridge, UK, ab186321	-	1:500
Goat anti-rabbit (Alexa fluor-555)	Abcam, Cambridge, UK, ab150078	-	1:750
Goat anti-mouse (Alexa fluor-488)	Abcam, Cambridge, UK, ab150113	-	1:750
Goat anti-rabbit HRP	Promega, Wisconsin W4018	1:15,000	-
Goat anti-mouse HRP	Jackson, 115-035-166	1:5000	
